# Effects of lifelong intake of lemon polyphenols on aging and intestinal microbiome in the senescence-accelerated mouse prone 1 (SAMP1)

**DOI:** 10.1038/s41598-019-40253-x

**Published:** 2019-03-06

**Authors:** Chikako Shimizu, Yoshihisa Wakita, Takashi Inoue, Masanori Hiramitsu, Miki Okada, Yutaka Mitani, Shuichi Segawa, Youichi Tsuchiya, Toshitaka Nabeshima

**Affiliations:** 1 Frontier Laboratories for Value Creation, SAPPORO HOLDINGS LTD., 10 Okatome, Yaizu, Shizuoka, 425-0013 Japan; 2 Foodtechnology Laboratories for Value Creation, SAPPORO HOLDINGS LTD, 1189-4 Nippa-cho, Kouhoku-ku, Yokohama 223-0057 Japan; 3 POKKA SAPPORO Food & Beverage Ltd., 45-2 Juniso, Kumanosho, Kitanagoya, Aichi 481-8515 Japan; 4 Fujita Health University, 1-98 Dengakugakubo, Kutsukake-cho, Toyoake, Aichi 470-1192 Japan; 5 NPO Japanese Drug Organization of Appropriate Use and Research, 3-1509 Omoteyama, Tanpaku-ku, Nagoya, 468-0069 Japan

## Abstract

Polyphenols have been examined for their beneficial effects on health, particularly in rodents, but their lifelong effects are unclear. Lemons (*Citrus limon*), containing lemon polyphenols (LPP), are widely consumed but the effects of LPP on aging are unknown. Therefore, we examined the effects of LPP on aging such as aging-related scores, locomotor activity, cognitive functions, and intestinal microbiome using senescence-accelerated mouse prone 1 (SAMP1) and senescence-accelerated resistant mouse 1 (SAMR1). All mice had *ad libitum* access to water (P1_water group, SAMR1) or 0.1% LPP (P1_LPP group). In the P1_LPP group, LPP intake prolonged the lifespan by approximately 3 weeks and delayed increases in aging-related scores (e.g., periophthalmic lesions) and locomotor atrophy. The P1_water group showed large changes in the intestinal microbiome structure, while the R1 and P1_LPP groups did not. The phylum Bacteroidetes/Firmicutes, which is associated with obesity, in the P1_water group was significantly lower and higher than that in the P1_LPP and R1 groups, respectively. Although the relative abundance of *Lactobacillus* significantly increased in both P1 groups with aging, the P1_LPP group showed a significantly lower increase than the P1_water group. Thus, lifelong intake of LPP may have anti-aging effects on both phenotypes and the intestinal environment.

## Introduction

Lemon fruit (*Citrus limon*) is one of the most widely consumed fruits, either directly or used in soft drinks, alcoholic drinks, and cooking. Lemons are rich in citric acid, vitamin C, and polyphenols, which confer various health benefits, such as the alleviation of fatigue^1^ and lipid-lowering effects^2,3^. Eriocitrin, the main lemon polyphenol (LPP), is a yellow and water-soluble antioxidant^2,4^ that is abundant in lemon juice and peel. Although the anti-aging effects of polyphenols have been suggested, few studies in rodents have been conducted until animal death, such as studies of tea and wine polyphenols^5,6^. However, humans are likely to consume the same habitual diets throughout their lifespan. Data obtained for a limited period may not reflect the beneficial effects or safety of a food. Thus, studies of the lifelong effects of foods are needed.

Senescence-accelerated mouse prone (SAMP) strains were established by Takeda *et al*.^7^. The SAMP substrain SAMP1 shows early deficits in age-associated pathological features such as senile amyloidosis, impaired immune response, and impaired motor function^8,9^ and has been widely used to analyse various antioxidants, such as reduced coenzyme Q10^10^. As an age-matched control, the senescence-accelerated resistant mouse 1 (SAMR1) is frequently used for comparison with SAMP1 to detect age-related changes.

Studies of the intestinal microbiome in the last decade have demonstrated its major effects on the host^11–13^. Turnbaugh *et al*.^14^ reported the relationship between the phylum Firmicutes/Bacteroidetes ratio and capacity to harvest energy from the diet. Additionally, changes in the intestinal microbiome throughout life have been reported^15,16^. In these contexts, changes in the microbiome involving aging may affect host health and function as markers of aging.

In this study, we investigated the lifelong effects of LPP in SAMP1 to evaluate healthy aging. From the perspective of welfare and management of animals, we did not sacrifice the mice, but evaluated their lifespan using non-invasive pharmacological methods and faeces to investigate the intestinal microbiota in P1 mice drinking LPP throughout life. For example, we examined aging-related phenotypes every month and cognitive functions and locomotor activity every 3 months. Additionally, the intestinal microbiome was evaluated.

This is the first report of the effects of LPP during the lifespan of mice.

## Results and Discussion

### Nutrition facts, polyphenols, and the anti-oxidative activities of LPP

LPP was obtained as a yellow solid. The nutritional components of LPP were as follows (w/w%): moisture content, 7.8; protein, 4.3; fat, 1.7; carbohydrates, 85.4; and ash, 0.8. The contents of structurally identifiable polyphenols contained in carbohydrates within LPP were as follows (w/w%): eriocitrin, 21.7; hesperidin, 3.5; eriodictyol-7-glycoside, 1.2; and eriodictyol, 0.4. Miyake *et al*.^17^ isolated and identified six flavanone glycosides and three flavone glycosides from lemon fruits (juice and peel). Eriocitrin showed the highest content of polyphenols in lemon fruits. Thus, we conformed that the main polyphenol component in LPP was eriocitrin.

The total phenol content in LPP according to the Folin-Ciocalteu method (reference compound: gallic acid)^18^ was 15.9% (w/w%). LPP showed anti-oxidative potency. The α-diphenyl-β-picrylhydrazyl (DPPH) free radical scavenging activity^19^ and oxygen radical absorbance capacity (ORAC)^20^ were 560 μmol Trolox equivalent (TE)/g and 5400 μmol TE/g, respectively. The anti-oxidative results of LPP appeared to contribute to the anti-oxidative activities and mechanisms.

We chose a dose of 0.1% LPP based on a previous study by Miyake *et al*.^21^. The previous study reported that after a 28-day feeding period in rats, the daily eriocitrin intake via pellets containing 0.2% eriocitrin was approximately 267 mg/kg/rat (e.g. body weight; 240 g, food intake; 32 g/day) and anti-oxidative effects were significantly increased. In our study, the mice were throughout life; therefore, we decreased the dose of LPP (containing 21.7% eriocitrin). An SAMP1 mouse (e.g. 30 g body weight) consumed 6 mL of 0.1% LPP solution each day, which corresponded to LPP 200 mg/kg/day (eriocitrin dose of 43 mg/kg/day).

### Food consumption, liquid consumption, body weight, and number of surviving mice in P1 mice drinking water or 0.1% LPP during the lifespan

**Figure 1:**
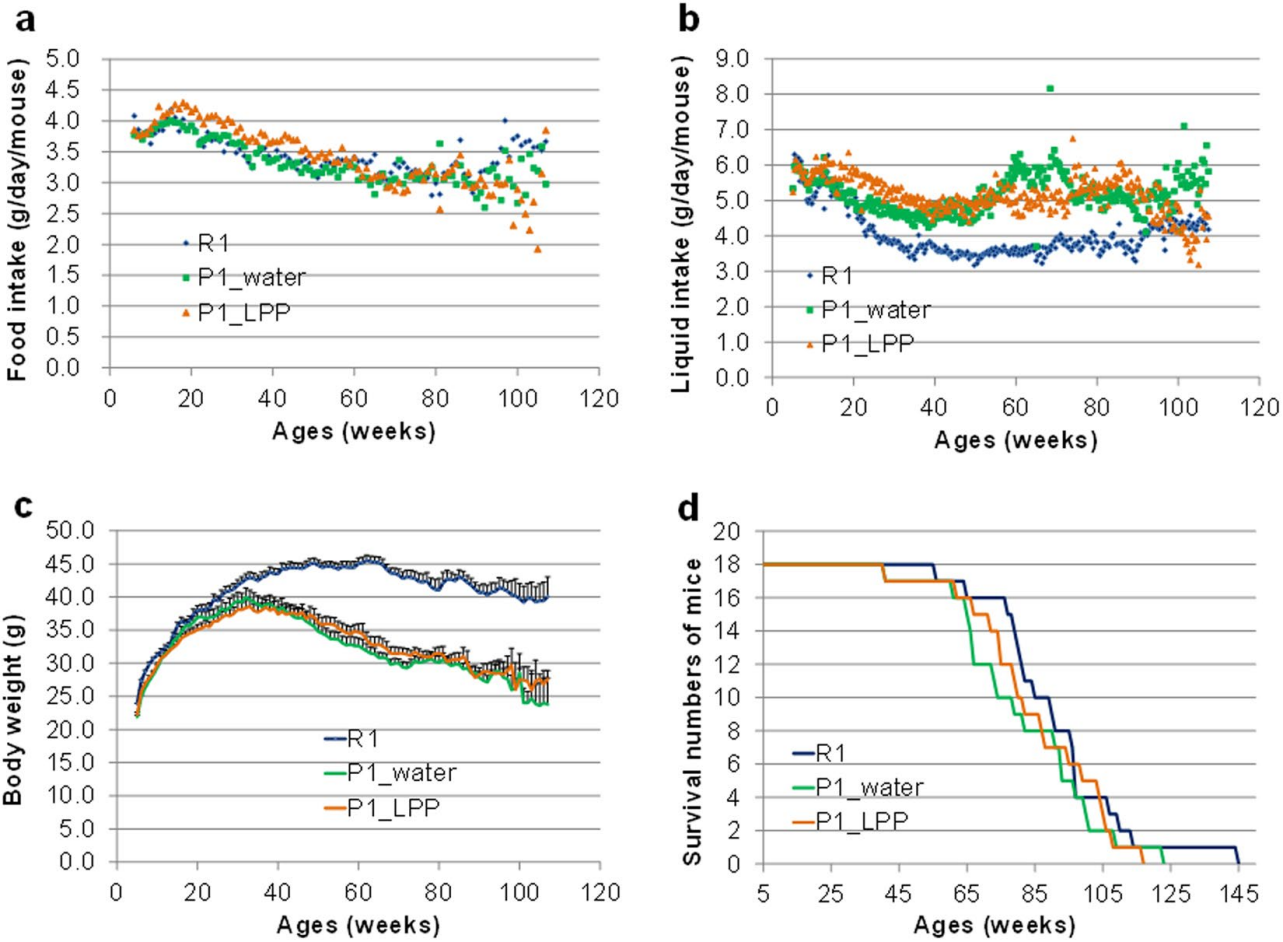
Food consumption, liquid consumption, body weight, and number of surviving mice in P1 mice drinking water or 0.1% LPP during the lifespan. (**a**) Food consumption (g/mouse/day). (**b**) Liquid consumption (g/mouse/day). (**c**) Body weight (g/mouse/day). (**d**) Number of surviving mice. dark blue line: R1 group; green line: P1_water group; orange line: P1_LPP.

The results for food consumption, liquid consumption, body weight, and number of surviving mice are shown in Fig. 1a–d. Food and liquid consumption is expressed as the consumption of each mouse per day, which was calculated by dividing the total consumption by the number of mice per cage.

Food consumption in all groups gradually decreased with age (Fig. 1a). There were no significant differences in food consumption, liquid consumption, and body weight between the P1_water and P1_LPP groups (Fig. 1a–c). Although spikes in liquid intake were occasionally observed (Fig. 1b), we predicted that the spikes were caused by contact between the top of the bottle nozzle and the cage paper. The contents of protein and fat in LPP were low (less than 5%). The caloric intake of a mouse in the P1_LPP group was 14.3 kcal from CRF-1 (approximately 4 g/day/mouse) and 0.02 kcal from LPP (approximately 6 mL/day/mouse). Thus, LPP accounted for only 0.16% of the total daily calories. We therefore assumed that the contributions of nutrient components and calories of LPP were negligible. In the R1 group, the liquid consumption was lower, but the average body weight was higher than in both P1 groups; however, food intake was nearly equal to that in the P1 groups (Fig. 1a–c). Body weight increased until 30 weeks old, but then gradually decreased in both P1 groups; body weight decreased from approximately 65 weeks old in the R1 group. We confirmed the differences in body weight and feed efficiency (body weight/food consumption) between the SAMR1 and SAMP1 groups in a previous report^22^. The results suggest that there was a major difference in metabolism between the two groups.

The mean lifespans of the groups were as follows: R1, 90.9 ± 4.7 weeks; P1_water, 81.8 ± 4.7 weeks; and P1_LPP, 85.0 ± 4.6 weeks (Fig. 1d). The average lifespan in the P1-LPP group was approximately 3 weeks longer than that in the P1-water group. At 74 weeks old, there was no significant difference in the numbers of surviving mice between the R1_group (16/18, number of surviving mice/number of mice used) and P1_LPP group (14/18) (*P* = 0.658), but mice in the R1 group tended to be larger than those in the P1_water group (10/18) (*P* = 0.060), as determined by Fisher’s exact test. Our findings showed that the lifelong intake of LPP extracts has positive effects over the lifespan.

### Changes in aging-related scores in P1 mice drinking water or 0.1% LPP during the lifespan

**Figure 2:**
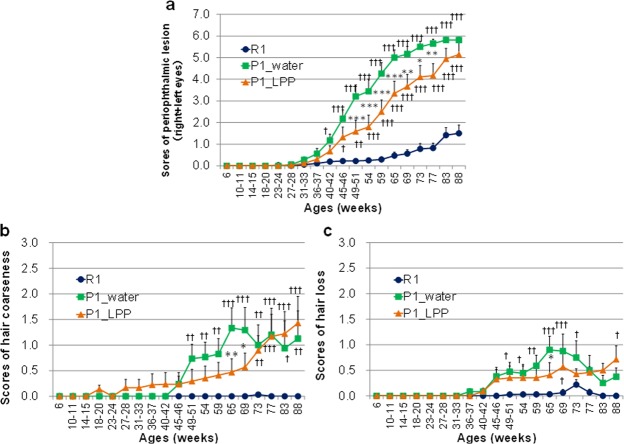
Changes in aging-related scores in P1 mice drinking water or 0.1% LPP during the lifespan dark blue: R1 group; green: P1_water group; orange: P1_LPP. (**a**) Periophthalmic lesion (Grade 0; no changes, Grade 1; catarrhal changes limited to the periophthalmic area or swelling of palpebra, Grade 2; catarrhal changes extending to nose, and Grade 3; catarrhal changes extending to further). (**b**) Hair coarseness (Grade 0; no coarseness, Grade 1; coarseness of less than an area of the head, Grade 2; coarseness of less than double the area of the head, Grade 3; coarseness of less than 3 times area of the head, and Grade 4; coarseness of over 3 times area of the head). (**c**) Air loss (Grade 0; neither loss or thinning of hair, Grade 1; loss of hair in less than an area of the head or thinning of hair in less than 1/2 of total area, Grade 2; loss of hair over one area of the head, less than 1/4 of total area or thinning of hair in more than 1/2 of total area, Grade 3; loss of hair in more than 1/4 in less than 1/2 of total area, and Grade 4; loss of hair in over 1/2 of total area). Between-group comparison among R1, P1_water, and P1_LPP groups by two-way ANOVA followed by Bonferroni’s *post hoc* test for multiple comparisons. ***P < 0.001, **P < 0.01, *P < 0.05; P1_water group vs. P1_LPP group. ^†††^*P* < 0.001, ^††^*P* < 0.01, ^†^*P* < 0.05; R1 group vs. P1_water or P1_LPP groups.

We examined the physical activity, skin conditions, eye inflammation, and spinal curvature in all groups nearly every month from 6 to 88 weeks old. There were significant differences in aging-related scores between the P1_water and P1_LPP groups in periophthalmic lesions, hair coarseness, and hair loss (Fig. 2a–c). Other non-significant data related to aging were not shown.

We performed two-way analysis of variance (ANOVA) followed by Bonferroni’s *post hoc* test to assess the effects of the group on aging-related scores. Group [*F*(2, 855) = 270.6, *P* < 0.001] and age [*F*(18, 855) = 76.2, *P* < 0.001] significantly affected the scores of periophthalmic lesions (Fig. 2a). There was a significant interaction between group and age [*F*(36, 855) = 13.2, *P* < 0.001]. Using the Bonferroni’s *post hoc* test, the scores of periophthalmic lesions as the sum of right and left eyes in R1 were significantly low compared to those of the P1_water group after the age of 40–42 weeks (40–42 weeks, *P* = 0.034; from 45–46 to 88 weeks old, *P* < 0.001) and those of the P1_LPP group after 45–46 weeks (45–46 weeks, *P* = 0.014; 49–51 weeks, *P* = 0.001; after 54 weeks, *P* < 0.001). From 69 to 77 weeks, the scores of periophthalmic lesions in the P1_LPP group were significantly lower than those in the P1_water group (from 49 to 65 weeks old, *P* < 0.001; 69 weeks old, *P* = 0.002; 73 weeks old, *P* = 0.010; 77 weeks old, *P* = 0.008).

In terms of hair coarseness, group [*F*(2, 855) = 40.5, *P* < 0.001] and age [*F*(18, 855) = 7.4, *P* < 0.001] showed significant effects (Fig. 2b). There was a significant interaction between group and age [*F*(36, 855) = 2.7, *P* < 0.001]. Using Bonferroni’s *post hoc* test, the scores of hair coarseness in R1 were significantly low compared to those of P1_water after 49–51 weeks of age (49–51 weeks, *P* = 0.008; 54 weeks, *P* = 0.005; 59 weeks, *P* = 0.003; 65 and 69 weeks, *P* < 0.001; 73 weeks, *P* = 0.003; 77 weeks, *P* < 0.001; 83 weeks, *P* = 0.014; 88 weeks, *P* = 0.003) and those of P1_LPP after 73 weeks of age (73 weeks, *P* = 0.003; from 77 to 88 weeks, *P* < 0.001). At 65 and 69 weeks old, the scores of hair coarseness in the P1_LPP group were significantly lower than those in the P1_water group (65 weeks, *P* = 0.003; 69 weeks, *P* = 0.029).

In terms of hair loss, group [*F*(2, 855) = 25.7, *P* < 0.001] and age [*F*(18, 855) = 6.9, *P* < 0.001] had significant effects on hair loss scores (Fig. 2c). There was a significant interaction between group and age [*F*(36, 855) = 1.7, *P* = 0.005]. Using Bonferroni’s *post hoc* test, the scores of hair loss in R1 were significantly low compared to those of the P1_water group from 49–51 to 73 weeks of age (49–51 weeks, *P* = 0.021; 54 weeks, *P* = 0.035; 59 weeks, *P* = 0.002; 65 and 69 weeks, *P* < 0.001; 73 weeks, *P* = 0.019), and those of the P1_LPP group at 69 weeks of age (*P* = 0.011) and 88 weeks of age (*P* = 0.008). At 65 weeks, the score of hair loss in the P1_LPP group was significantly lower than that in the P1_water group (65 weeks, *P* = 0.014).

In the R1 group, hair coarseness and hair loss were not changed by aging, which retained a youthful appearance (Fig. 2b,c). In contrast, hair aging rapidly progressed in the SAMP1 groups from approximately 40 weeks old. However, aging scores in the P1_LPP group were significantly lower than those in the P1_water group at 65–69 weeks old. For aging associated with periophthalmic lesions, aging in the R1 group occurred much more slowly than that in both P1 groups (Fig. 2a) and aging scores in the R1 group were one-third lower than those in the P1 groups, even at 88 weeks old. These results suggest that LPP delays aging, such as periophthalmic lesions, hair coarseness, and hair loss.

A significant decrease in the levels of lipid antioxidants and hydrophilic antioxidant carnosine was observed in the brain of SAMP1, which is characterized by accelerated accumulation of senile features, compared to SAMR1^23^. Antioxidative compounds delay the increase in aging scores in SAMP1^8^. The main polyphenol component of LPP, eriocitrin, is metabolized by intestinal bacteria and then absorbed to induce antioxidative activity in the plasma^24^. In this experiment, the P1_LPP group may have maintained a higher antioxidative activity by consuming 0.1% LPP water rather than tap water throughout life. One possible mechanism by which LPP prevents aging is via antioxidative activity against oxidative stress in SAMP1.

### Changes in locomotor activities in P1 mice drinking water or 0.1% LPP during the lifespan

**Figure 3:**
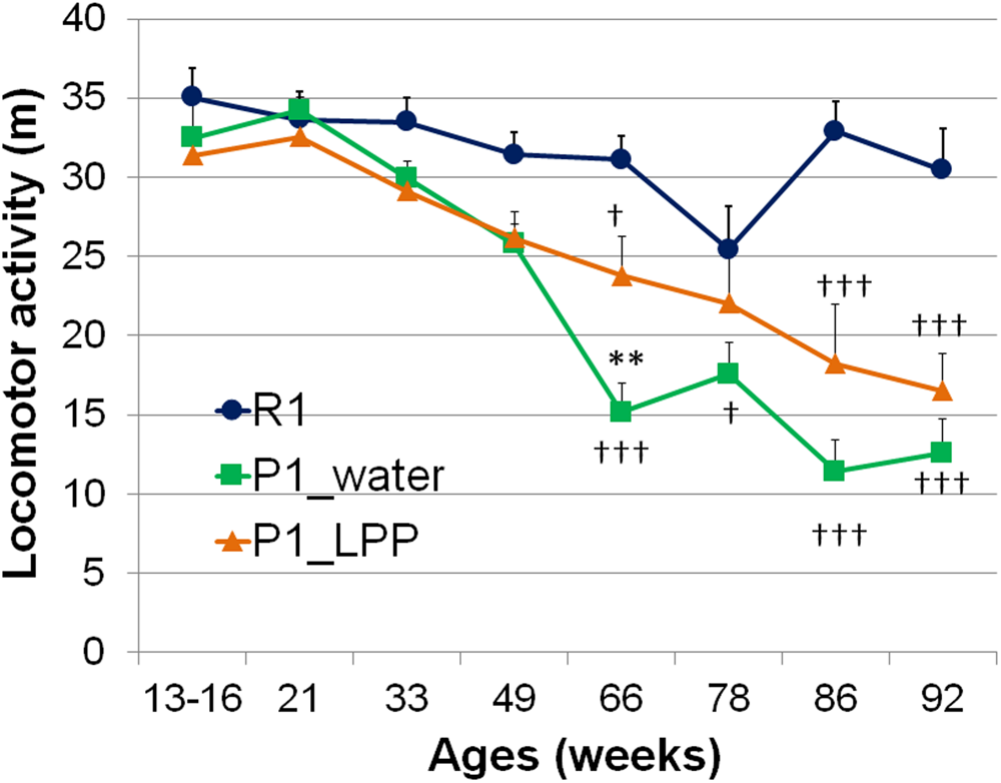
Changes in locomotor activities in P1 mice drinking water or 0.1% LPP during the lifespan. dark blue: R1 group; green: P1_water group; orange: P1_LPP. Between-group comparison among R1, P1_water, and P1_LPP groups by two-way ANOVA followed by Bonferroni’s *post hoc* test for multiple comparisons. *P < 0.05; P1_water group vs. P1_LPP groups. ^†††^*P* < 0.001, ^†^*P* < 0.05; R1 group vs. P1_water group or P1_LPP group.

To ensure healthy aging, it is necessary to maintain locomotor activity to avoid frailty, sarcopenia, and a bedridden state, which are caused by not only low physical activity and a lack of protein nutrition but also an inflammatory profile and oxidative stress^25,26^. We examined the changes in locomotor activity with aging nearly every 3 months from 13–16 to 92 weeks old (Fig. 3). Two-way ANOVA followed by Bonferroni’s *post hoc* test revealed that locomotor activities in the R1 group were significantly higher than those in the P1_water group at 66 (*P* < 0.001), 78 (*P* = 0.028), 86 (*P* < 0.001), and 92 weeks old (*P* < 0.001) and in the P1_LPP group at 66 (*P* = 0.022), 86 (*P* < 0.001), and 92 weeks old (*P* < 0.001). Moreover, there was a significant difference between the P1_water and P1_LPP groups at 66 weeks old (*P* = 0.005). Locomotor activity in the R1 group was nearly unchanged with aging, while those in both P1 groups were significantly decreased with aging. Aoyama *et al*.^9^ reported that cerebellar Purkinje cells in 4-month-old SAMP1 mice persistently express tyrosine hydroxylase, the overexpression of which is associated with motor dysfunction. In this experiment, locomotor impairment in SAMP1 appeared at 33 weeks old and deteriorated with aging. Interestingly, long-term LPP intake slowly prevented locomotor atrophy with aging in the P1_LPP group compared to in the P1_water group at 66 weeks old (Fig. 3).

We attempted to measure the levels of 8-hydroxydeoxyguanosine (8-OHdG)^27^, a biomarker of oxidative DNA damages in the urine at 68–71 weeks in this study. However, this measurement was not possible because the volumes of urine in SAM mice were too small. In many urine samples, 8-OHdG was below the measurable limit after dilution (data not shown). Miyake *et al*. reported^21^ that eriocitrin significantly deceased 8-OHdG in the urine of diabetic rats after a 28-day feeding period. Moreover, administration of eriocitrin increased antioxidant activity in the plasma^24^. Further, Ferreira *et al*.^28^ reported that citrus flavanones (hesperidin, eriocitrin, and eriodictyol) increase the serum total antioxidant capacity and restrain elevations in inflammatory cytokines, such as interleukin-6 and macrophage chemoattractant protein-1. Therefore, the anti-aging effects of LPP may delay not only increases in aging scores (Fig. 2a–c), such as periophthalmic lesions, but also decreases in locomotor activity (Fig. 3) via antioxidative and anti-inflammatory activities throughout the body.

### Changes in object recognition (long-term object memory) and spatial recognition (short-term location memory) in P1 mice drinking water or 0.1% LPP during the lifespan

**Figure 4:**
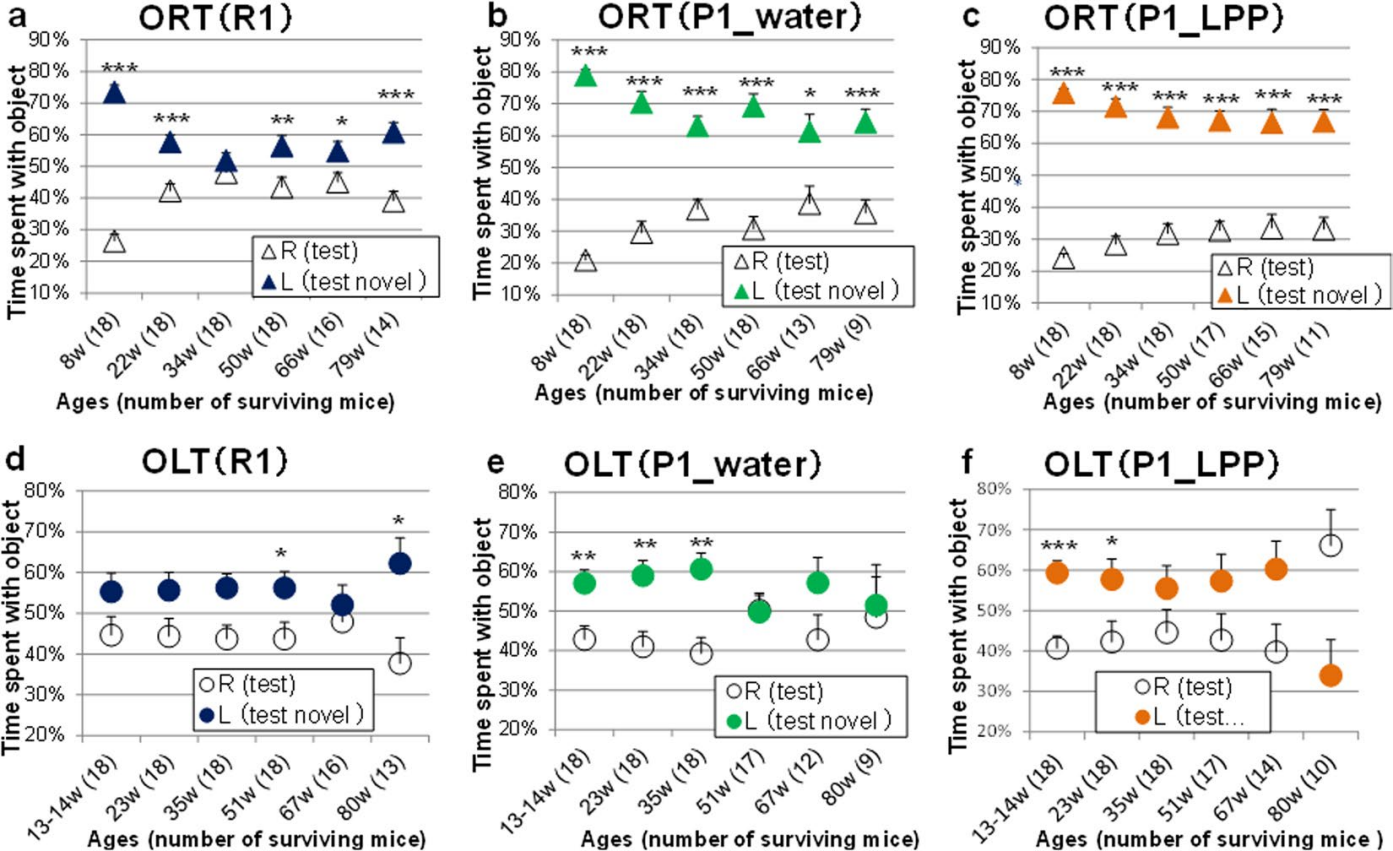
Changes in object recognition (long-term object memory) and spatial recognition (short-term location memory) in P1 mice drinking water or 0.1% LPP during the lifespan. Recognition indexes during the test session of the ORT: (**a**) R1 group, (**b**) P1_water group, (**c**) P1_LPP group. Recognition indexes during the test session of the OLT: (**d**) R1 group, (**e**) P1_water group, (**f**) P1_LPP group Δ: familiar object; ▲: novel object (dark blue: R1 group; green; P1_water group; orange: P1_LPP) ○: familiar location object; ●: novel location object (dark blue: R1 group; green; P1_water group; orange: P1_LPP). The numbers in parentheses show the number of surviving mice at the different ages. ***P < 0.001, **P < 0.01, *P < 0.05; by unpaired *t*-test.

SAMP1 is not typically used as an early cognitive deficit model, similar to SAMP8 and SAMP10^5,8,29^. We investigated whether moderate/lifelong LPP intake protects against age-related impairment of recognition in an object recognition test (ORT) and object location test (OLT). The recognition indices of familiar and novel objects during the test phase in ORT for the R1, P1_water, and P1_LPP groups are illustrated in Fig. 4a–c, respectively. The recognition indices of the familiar and novel locations during the test phase in OLT for R1, P1_water, and P1_LPP groups are illustrated in Fig. 4d–f, respectively. During the training phase, the recognition indices were nearly 50% and no significant difference in the preference was observed between the left and right objects (data not shown), as the two objects were identical.

In the ORT during the test phases from 8 to 79 weeks old (i.e., nearly the entire life), the recognition index for the novel object (film case) was significantly higher than that for the familiar object (golf ball) in both the P1_water and P1_LPP groups (*P* < 0.05; Fig. 4b,c). Although the preference for the novel object in the R1 group was significantly higher than that for the familiar object (*P* < 0.05), except at 34 weeks old, the recognition indices of the R1 group (novel object: less than 60% except at 8 weeks old) were lower than those of both P1 groups (novel object: more than 60%), despite that SAMR1 is a senescence-accelerated resistant mouse (Fig. 4a). During breeding of these mice, we found differences in behaviour between SAMR1 and SAMP1 in the home cages. Unlike SAMP1, SAMR1 always made a deep nest using sliced paper in the cage and hid in the nest. Therefore, SAMR1 showed more fear than SAMP1 towards objects in the ORT and OLT. The total access time to objects in the ORT during the training session in the R1 group was approximately 50% shorter than that in the P1 group (data not shown). The lower recognition indices in the R1 group may be related to the short approach time to the objects in the ORT during the training session.

In the OLT during the test phase from 9 to 80 weeks old, regarding the recognition index of familiar and novel locations, the mice recognized the novel position significantly more than the familiar location from 13–14 to 35 weeks old in the P1_water group (*P* < 0.05; Fig. 4e) and from 13–14 to 23 weeks old in the P1_LPP group (*P* < 0.05; Fig. 4f), but not at 80 weeks old. In the R1 group, there were no significant differences between the familiar and novel positions from 13–14 to 35 weeks old, but significant differences were identified at 51 and 80 weeks old. The disorder of spatial recognition in the OLT (Fig. 4d–f) preceded the impairment of object recognition in the ORT (Fig. 4a–c), as we reported previously^30^. In the comparison of P1 groups, the recognition indices for a novel location were significantly higher than those for a familiar location from 13–14 to 35 weeks old in the P1_water group, but only from 13–14 to 28 weeks old in the P1_LPP group (Fig. 4e,f). However, from 35 to 67 weeks old in the P1_LPP group, the indices for a novel location were higher than those for a familiar location (no significance) and tended to be higher at 67 weeks old (*P* = 0.064), but not in the P1_water group (*P* = 0.137). The results of the OLT showed that location-related memory in the P1_LPP group was not worse than that in the P1_water group. Based on the recognition ability, we examined the temporary changes that occurred during the lifespan rather than a few time points within a certain period to avoid misleading results.

Compared to the P1_water groups, the recognition indices in the OLT in the R1 group were lower, as observed in the ORT; however, recognition of the location of a novel object was significantly better than that for a familiar location at 80 weeks old (Fig. 4d). We assumed that the R1 group was not inferior to the P1_groups, as they approached objects in a short time in the training session, similar to that in the ORT. The cognitive effects of long-term LPP intake were weaker than those on other phenotypes.

The activities of antioxidant enzymes in the brain of SAMP1 are significantly lower than those in SAMR1^22^. Oxidative stress may cause disorder in spatial recognition in SAMP1. Tea polyphenols, epigallocatechin gallate with antioxidant activity, and its metabolite, 5-(3′,5′-dihydroxyphenyl)-γ-valerolactone can pass the blood–brain barrier (BBB) and directly affect the memory-retarding activity in aged mice^29^. Although LPP has strong antioxidant activity^21^, it has a low ability to pass the BBB because of its glycoside form. Eriocitrin is metabolized to eriodictyol, methylated eriodictyol, 3,4-dihydroxyhydrocinnamic acid, and their conjugates in the plasma and urine^24^. Further studies are needed to determine whether metabolites can pass the BBB and directly affect cognitive functions.

### Changes in intestinal microbiome at 19 and 70 weeks old in P1 mice drinking water or 0.1% LPP

**Figure 5:**
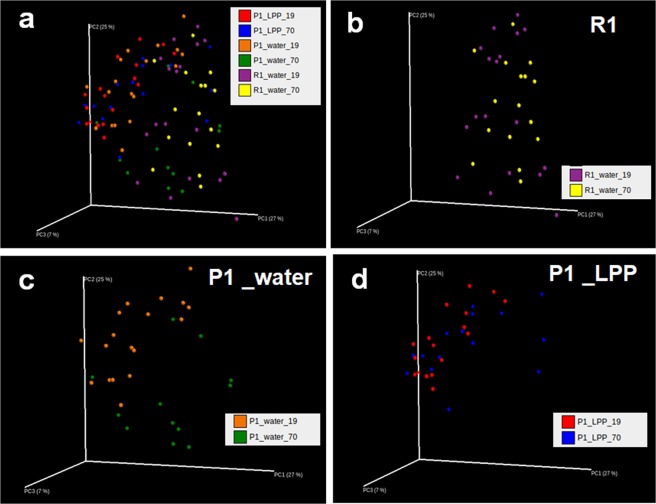
Changes in intestinal microbiome by UniFrac analyses at 19 and 70 weeks old in P1 mice drinking water or 0.1% LPP. All group, (**b**) R1 group, (**c**) P1_water group, (**d**) P1_LPP group. R1 group, purple: 19 weeks old; yellow: 70 weeks old. P1_water group, orange: 19 weeks old; green: 70 weeks old. P1_LPP group, red: 19 weeks old; blue: 70 weeks old.

UniFrac analysis is an effective distance metric for microbial species^31^ and visually expresses the composition of bacterial species at a specific site. Initially, the overall structure of the intestinal microbiome was evaluated by UniFrac analysis (Fig. 5a–d). At 19 weeks old, the structure of the intestinal microbiome in the R1 group (Fig. 5b) differed from that in both P1 groups (Fig. 5c,d). Moreover, the microbiome at 19 weeks old differed from that at 70 weeks old in the P1_water group (Fig. 5c), but not in the R1 and P1_LPP groups (Fig. 5b,d). These results suggest that LPP intake maintains the intestinal environment against aging.

**Figure 6:**
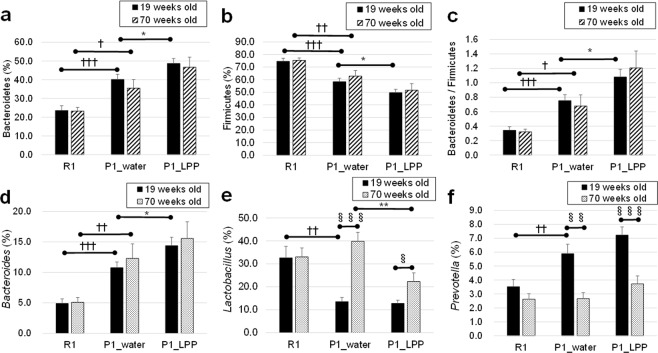
Changes in intestinal microbiome phylum and genus families at 19 and 70 weeks old in P1 mice drinking water or 0.1% LPP. (**a**) Bacteroidetes (phylum), (**b**) Firmicutes (phylum), (**c**) Bacteroidetes (phylum)*/*Firmicutes (phylum), (**d**) *Bacteroides* (genus), (**e**) *Lactobacillus* (genus), and (**f**) *Prevotella* (genus). *P < 0.05, **P < 0.01; P1_water group vs. P1_LPP group by unpaired *t*-test. ^§^*P* < 0.05, ^§§^*P* < 0.01, ^§§§^*P* < 0.001; 19 weeks old vs. 70 weeks old by unpaired *t*-test. ^†^*P* < 0.05, ^††^*P* < 0.01, ^†††^*P* < 0.001; R1 group vs. P1_water group by unpaired *t*-test.

Subsequently, the microbiome composition at the phylum level was evaluated. In between-group comparison of SAMR1 with SAMP1, the level of phylum Bacteroidetes in the P1_water group was significantly higher than that in the R1 group (Fig. 6a) at both 19 (*P* < 0.001) and 70 weeks old (*P* = 0.012), while the level of phylum Firmicutes was significantly lower (Fig. 6b) at 19 (*P* < 0.001) and 70 weeks old (*P* = 0.008). Furthermore, the level of Bacteroidetes/Firmicutes in the R1 group was significantly lower than that in the P1_water group (Fig. 6c) at 19 (*P* < 0.001) and 70 weeks old (*P* = 0.018). Recently, links between certain diseases and the intestinal microbiome have been suggested^32^. Human gut microbes are associated with obesity; a lower level of Bacteroidetes and higher level of Firmicutes have been detected in obese subjects^33^. In the R1 and P1_water groups, the difference in body weight between these groups gradually increased, becoming significant at 33 weeks old onwards (the R1 group had a higher weight than the SAMP1 group) (Fig. 1c), despite similar levels of intake of the same food (Fig. 1a). This may be explained by the composition of the SAMR1 intestinal microbiome.

In between-group comparison of P1_water with P1_LPP, the ratio of the phylum Bacteroidetes in the P1_LPP group was significantly higher (*P* = 0.033; Fig. 6a), while that of Firmicutes was significantly lower (*P* = 0.031; Fig. 6b). Furthermore, the level of Bacteroidetes/Firmicutes was significantly higher than that in the P1_water group (*P* = 0.023; Fig. 6c) at 19 weeks old; this difference was also observed at 70 weeks old, but was not significant (*P* = 0.088). However, the body weight was nearly the same between P1_LPP and P1_water groups, indicating that the microbiome is influenced by the feed (high-fat feed or not) and host status (intestinal absorption) in SAMP1.

Genus-level differences in the microbiome among the 3 groups were evaluated. The level of *Bacteroides* (phylum Bacteroidetes) in the P1_water group was significantly higher than that in the R1_water group at 19 (*P* < 0.001) and 70 weeks old (*P* = 0.004), but lower than that in the P1_LPP group at 19 weeks old (*P* = 0.038), as shown in Fig. 6d.

An increase in *Lactobacillus* strains associated with aging has been reported in human microbiota^15^. In within-group comparison with aging in each bacterial genus, *Lactobacillus* (Firmicutes phylum) significantly increased with aging in the P1_groups (P1_water group, *P* < 0.001; P1_LPP group, *P* = 0.016; Fig. 6e), but this was not observed for the R1_group (*P* = 0.860; Fig. 6e). Interestingly, the level of *Lactobacillus* at 70 weeks old in the P1_LPP group was significantly lower than that in the P1_water group (*P* = 0.004), indicating that LPP intake delayed the aging-related increase in *Lactobacillus*.

Eriocitrin is metabolized by intestinal bacteria, after which the metabolites are absorbed^24^, increasing anti-oxidative activities in the plasma^24^ and decreasing oxidative stress^21^. We predicted that the anti-aging effects of LPP are developed through the intestinal microbiome both directly and indirectly. We previously reported that the intestinal microbiota varies between breeder companies because of their different breeding environments, even in the same strain of mice^34^. In this aging study, we used the same breeding chamber and same type of cages. Therefore, the changes in the microbiota appear to be related to aging rather than the breeding environment.

Significant decreases with aging were observed in *Parabacteroides* (P1_water and P1_LPP, *P* < 0.001), *Prevotella* (P1_water, *P* = 0.001; P1_LPP, *P* < 0.001, Fig. 6f), *Oscillospira* (P1_water, *P* = 0.047), and *Ruminococcus* (R1, *P* = 0.037; P1_water, *P* = 0.020) (data not shown except for *Prevotella* Fig. 6f). Arumugam *et al*.^35^ reported that *Prevotella*, *Bacteroides*, and *Ruminococcus* were dominant in the intestinal microbiome and referred to as the three “enterotypes.” Enterotypes are linked with long-term diets, particularly protein, fat, and carbohydrates^36^. Remarkable changes in key microbiota for the human enterotype^35,36^ were observed by the LPP intake and aging in this study.

Our results suggested that lifelong intake of LPP has anti-aging effects not only on the host health status but also on the intestinal environment. Recently, Henning *et al*.^37^ reported that green and black tea polyphenol diets decrease weight gain and result in a decrease in cecum Firmicutes and increase in Bacteroidetes. Additional studies are needed to clarify whether intestinal or phenotypic changes occur at first during the intake of polyphenols.

## Conclusions

In the P1_LPP group, the average lifespan was approximately 3 weeks longer, and increases in aging-related scores (e.g. periophthalmic lesions) and locomotor atrophy were delayed compared to in the P1_water group. Additionally, possible aging-related changes in the intestinal microbiomes, such as for the genus *Lactobacillus*, were restricted by LPP intake. These results suggest that LPP has anti-aging effects not only on host health but also on the intestinal environment.

Evaluating lifelong intake of food is important for detecting the effects on human and animals because they are likely to consume habitual diets throughout their lifespan. Food habits may exert a large influence on the host.

## Materials and Methods

### Lemon polyphenol (LPP) extract

Lemon polyphenols (LPP) from lemon peel were obtained as described in a previous report^38^. LPP was subdivided into small packages and stored at −30 °C until usage. We freshly prepared 0.1% (w/v) LPP by dissolution in tap water and filtering through a Stericup™ (0.22 µm, SCGPU02RE; Merck Millipore, Billerica, MA, USA) twice per week.

### Animals

Thirty-six SAMP1 male mice aged 5 weeks and 18 SAMR1 (Japan SLC, Inc., Hamamatsu, Japan) were acclimated to the animal facility. The floor of the cage was covered with sliced paper, Palmas μ® (Material Research Center, Tokyo, Japan), which was changed every week. SAMR1 mice were group-housed (six mice per cage) with free access to tap water. The SAMP1 mice were also group-housed (six mice per cage) with free access to tap water (water group, *n* = 18) or 0.1% (w/v) LPP [LPP group; water until 8 weeks of age, 0.1% (w/v) LPP from 9 weeks of age, *n* = 18].

All mice were provided *ad libitum* access to standard chow (CRF-1; Charles River Laboratories, Yokohama, Japan). The animal facility was maintained at 23 °C ± 1 °C with 55% humidity and a 12-h/12-h light/dark cycle. We used the same breeding chamber (EBAC-L^®^, CLEA Japan, Inc., Tokyo, Japan) and same type of cages (Clean S-PSF^®^, CLEA Japan Inc., Tokyo, Japan) in this aging study.

All experiments were approved by the Institutional Animal Care and Use Committee of SAPPORO BREWERIES LTD. (permit number 2014-006) following the Guidelines for the Proper Conduct of Animal Experiments on the Science Council of Japan. All experimental protocols and animal procedures were conducted in accordance with the approved guidelines.

### Food consumption, liquid consumption, body weight, and survival analysis

Food consumption and liquid consumption per cage were recorded every week and three times every week, respectively. These values were expressed as one mouse’s consumption per day, which was calculated by dividing the total consumption per cage by the number of mice in that cage. The body weight of each mouse was examined every week.

### Grading aging-related scores

During the lifespan of all mice, we examined the changes in grading scores, such as those for skin and hair conditions (glossiness, hair coarseness, and hair loss), ulcers, eyes (cataract, periophthalmic lesions, opacity of cornea, and ulcer of cornea), and skeleton (spinal curvature) nearly every month from 6 weeks to 88 weeks old, as reported previously^39^.

### Object recognition test (ORT) and object location test (OLT)

The novel ORT and OLT for spatial cognition are non-invasive and conducted under conditions similar to those used for human cognitive assessment^40^.

We used the same boxes [300 × 300 × 350 mm (D × W × H); Brain Science Idea, Inc., Osaka, Japan] covered with black plastic tetra-laterally for both the ORT and OLT. We measured cognitive functions by ORT and OLT as previously reported^30^. For ORT objects, white golf balls (43-mm diameter) were used as training objects and a white film case [29 mm (diameter) × 50 mm (high)] as the novel object. For OLT objects, apple-shaped wooden blocks without colouring (31-mm width and 50-mm height of main body and 15-mm height of stem end) were used. In the ORT and OLT training phases, mice were placed in the box and allowed to freely access the two objects for 10 min, whereas in the test phase, the free access time was 10 min for the ORT and 5 min for the OLT. The interval between the training and test phases was 24 h for the assessment of long-term memory during the ORT, but 2 h for short-term memory during the OLT because SAMR1 and SAMP1 mice could not maintain long-term spatial memory for the OLT (data not shown).

The ORT experiments were conducted at 8, 22, 34, 50, 66, and 79 weeks old and the OLT experiments were conducted at 13, 14, 23, 35, 51, 67, and 80 weeks old to evaluate cognitive function. In the training phase, the recognition index of the right and left objects for each mouse was expressed as the ratio of the amount of time spent exploring object left A (time left A × 100)/(time left A + time right A) and amount of time spent exploring object right A (time right A × 100)/(time left A + time right A) for both ORT and OLT. During the test phase, the recognition index for each mouse was expressed as the ratio of the amount of time spent exploring the familiar object A (time A × 100)/(time A + time B) and amount of time spent exploring the novel object B (time B × 100)/(time A + time B) for the ORT or familiar object A (time A × 100)/(time A + time A′) and amount of time spent exploring novel location object A′ (time A′ × 100)/(time A + time A′) for the OLT. Differences between recognition indices of left and right (or novel location) objects were assessed by using the unpaired *t*-test for ORT (OLT) in each phase.

### Locomotor activity

Locomotor activity for 10 min (staying time and locomotor distance in the ORT box) was quantified using the ANY-maze Video Tracking System (Stoelting Co., Wood Dale, IL, USA). Illumination was provided at 25 lux. A black patch was attached to the back of each mouse to enable tracking in the ORT box with a white-coloured floor.

### Analysis of intestinal microbiome

Fresh faeces (approximately 100 mg) were collected, stored at −30 °C, and used to analyse the intestinal microbiome. Bacterial DNA was isolated as described by Matsuki *et al*.^41^ with some modifications. Briefly, the bacterial suspension was treated with lysis buffer at 70 °C for 10 min in a water bath and vortexed vigorously for 60 s with a Micro Smash MS-100 (Tomy Digital Biology Co., Ltd., Tokyo, Japan) at 4,000 rpm.

Primers for amplification of the V1 and V2 regions of the 16S rRNA gene reported by Kim *et al*.^42^ were used with some modifications^34^. The following primers were used: forward primer (5′-CCATCTCATCCCTGCGTGTCTCCGACTCAGNNNNNNNNNNGTagrgtttgatymtggctcag-3′) containing the Ion PGM sequencing primer A-key, a unique erroneous 10–12-base pair barcode sequence (indicated with N), ‘GT’ spacer, and 27Fmod (agrgtttgatymtggctcag); and reverse primer (5′-CCTCTCTATGGGCAGTCGGTGATtgctgcctcccgtaggagt-3′) containing the Ion PGM primer P1 and 338 R (tgctgcctcccgtaggagt). PCR was performed in a 25-μL reaction volume. Each reaction mixture contained 22.5 μL of platinum PCR mix, 2 μL of template DNA (approximately 4 ng), and 0.5 μL of 10-μM primer mix. The amplification reaction was carried out in the Veriti Thermal Cycler (Applied Biosystems, Foster City, CA, USA) by using the following program: 3 min at 94 °C followed by 25 cycles of 30 s each at 94 °C, 45 s at 55 °C, and 1 min at 68 °C. After each reaction, the mixture was purified using a PureLink Quick PCR Purification Kit (Invitrogen, Carlsbad, CA, USA); the concentration of each purified sample was measured using a Qubit 2.0 fluorometer (Life Technologies, Carlsbad, CA, USA). Purified samples were mixed at equal concentrations. The mixed sample was visualized by electrophoresis on 2% agarose gel and purified by gel extraction using a FastGene Gel/PCR Extraction Kit (Nippon Gene Co. Ltd., Tokyo, Japan). Emulsion PCR and sequencing were carried out by Ion PGM sequencing systems (Life Technologies).

After sequencing, the obtained reads were analysed by the QIIME pipeline (http://qime.org/) for the assignment of taxonomic classification. The reads that contained precise primer sequences (27Fmod and 338R) were selected, and those with an average quality value >20 were used for further analysis. The reads were grouped into operational taxonomic units (OTUs) with a sequence identity threshold of 97%; chimeric OTUs were removed by using ChimeraSlayer. UniFrac distance analysis was performed, and the proportion of the intestinal microbiome at the phylum and genus levels was determined by RDP (Ribosomal Database Project) classifier using the greengenes database (gg_13_8_otus/taxonomy/97_otu_taxonomy). The values are presented in the relative abundance of microbiota.

### Statistical analyses

SPSS software 10.0.7J for Windows (SPSS, Inc., Chicago, IL, USA) was used for all statistical analyses. Data in the text and figures are presented as the mean ± standard error of the mean. For locomotor activity and aging scores, between-group comparisons were performed by two-way analysis of variance (ANOVA) followed by Bonferroni’s *post hoc* test for multiple comparisons. Between-group comparisons (between bacterial species) were performed by unpaired *t*-tests. In all analyses, a *P*-value < 0.05 was considered statistically significant.
